# Tumor genomic alterations in severe-combined immunodeficiency bare-lymphocyte syndrome genes are associated with high mutational burden and disproportional neo-antigen rates

**DOI:** 10.1186/s40425-019-0584-2

**Published:** 2019-05-07

**Authors:** Yu Wang, Douglas B. Johnson, Steve Lu, Luis A. Diaz, Yaomin Xu, Justin M. Balko

**Affiliations:** 1 Department of Biostatistics, Vanderbilt University Medical Center, Nashville, TN 37232 USA; 2 Division of Hematology and Oncology, Department of Medicine, Vanderbilt University Medical Center, Nashville, TN 37232 USA; 3 Breast Cancer Research Program, Vanderbilt University Medical Center, Nashville, TN 37232 USA; 4 Johns Hopkins University School of Medicine, Baltimore, MD 21287 USA; 5 Memorial Sloan Kettering Cancer Center, New York, NY 10065 USA; 6 Center for Quantitative Sciences, Vanderbilt University Medical Center, 2200 Pierce Ave, 777 PRB, Nashville, TN 37232–6307 USA

**Keywords:** Bare lymphocyte syndrome, Severe combined immunodeficiency syndrome, Antigen presentation, Neoantigens, Cancer, Immunotherapy

## Abstract

The progression of cancer requires mutational adaptation to permit unrestrained proliferation. A fraction of cancer mutations are oncogenic drivers, while others are putative ‘passengers’ that do not contribute to oncogenesis. However, altered peptides arising from passenger mutations may bind MHCs and activate non-self immunologic signals (i.e. neoantigens), thus requiring immunoediting for cancer persistence. Disruption of antigen processing machinery in tumor cells may diminish this requirement. Here, we show that rare mutations in antigen processing machinery are associated with high mutational burden and increased predicted neoantigen load, providing insights into the mechanisms of high mutation burden in some patients.

**Electronic supplementary material:**

The online version of this article (10.1186/s40425-019-0584-2) contains supplementary material, which is available to authorized users.

## Background

Cancer-expressed neoantigens are a major determinant of immunological activity and response to immunotherapy [1–9]. Tumor types with high tumor mutational burden (TMB), generally corresponding to high predicted neoantigen loads (e.g. *POLE*-mutant [10–12] and mismatch repair deficient/microsatellite-instable tumors; MSI) [13] demonstrate less aggressive disease courses and higher responses to immunotherapies targeting CTLA-4 and PD-1/L1 checkpoints [14–16]. Recently, it has been demonstrated that tumors with acquired resistance to immunotherapies can undergo immunoediting (genomic loss of neoantigens [1]) or lose key antigen presentation molecules such as beta-2-microglobulin (B2M) [17], a prerequisite for peptide-major histocompatibility complex class I (MHC-I) formation at the cell surface. Thus, immunoediting and genomic alterations in antigen presentation may work together or independently to circumvent adaptive immunity. However, other mechanisms whereby tumors may avoid T cell recognition through disruption of antigen presentation have not been thoroughly explored. Further, the process whereby tumors acquire high mutation burden while avoiding immunoediting is not well characterized in many cases.

Taking a lesson from severe combined immunodeficiency (SCID) hereditary disorders in humans, we asked whether somatic alterations in genes involved in bare lymphocyte syndrome (BLS) may be a possible mechanism for tumors to circumvent adaptive immunity and antigen presentation. BLS is a rare hereditary immunodeficiency syndrome characterized by loss-of-function germline alterations in MHC machinery (i.e. those molecules required for expression of MHC as well as peptide/antigen loading and transport) [18]. Type I BLS is caused by germline alterations in *TAP1*, *TAP2*, or *TAPBP*, genes encoding proteins involved in transport of peptides into the endoplasmic reticulum for loading onto MHC-I. Type II BLS results from alterations in *CIITA*, *RFX5*, *RFKAP*, or *RFXANK*, which are required for transcription of MHC-II, leading to lymphocytes (and other cell types) which lack MHC-II antigen presentation. Both Type I and Type II BLS lead to poor or nonexistent antigen presentation (via MHC-I or MHC-II) and immunodeficiency (i.e. ‘immune ignorance’) [18], resulting in susceptibility to infections and early mortality.

It is conceivable that tumor cells could somatically alter these genes in order to avoid antigen presentation synonymous to Type I or II BLS. Interestingly, somatic variants in Type-II BLS genes identified in a subset of gastric cancers were found to be associated with lack of recurrence [19]. To explore BLS genes as a target for somatic disruption of antigen presentation, we investigated four cancer types with high TMB in TCGA (colorectal, melanoma, gastric and uterine). We predicted MHC-I neoantigens using a computational approach with publicly available next generation sequencing databases. High mutational burden and increased neoantigen load are associated with rare mutations in BLS genes. In addition, immune surveillance is believed to be a selection force shaping cancer evolution, potentially acting to remove neoantigen-carrying clones by purifying (negative) selection. Consistently, reduced purifying selection on putative neoantigen-carrying genes are detected in patients with Type I BLS gene alterations, suggesting loss of immunoediting due to disrupted antigen presentation. Our findings show an insight into the mechanisms of high mutation burden, and potentially a loss of immunoediting in patients with somatic BLS alterations.

## Results and discussion

We hypothesized that somatic alteration of BLS genes in tumors could abrogate the necessity of immuno-editing by reducing antigen presentation. To test this hypothesis, we determined the rate of mutations in Type I and Type II BLS genes across human tumor datasets in cBioPortal [20]. We identified sporadic mutations in BLS genes (MHC-I and MHC-II) across cancer types, some of which were recurrent (Additional file 1: Figure S1). We specifically focused on tumor types with single-nucleotide variants and indels. Interestingly, we noted a high preponderance of truncating and early-stop codon alterations in BLS genes.

**Fig. 1:**
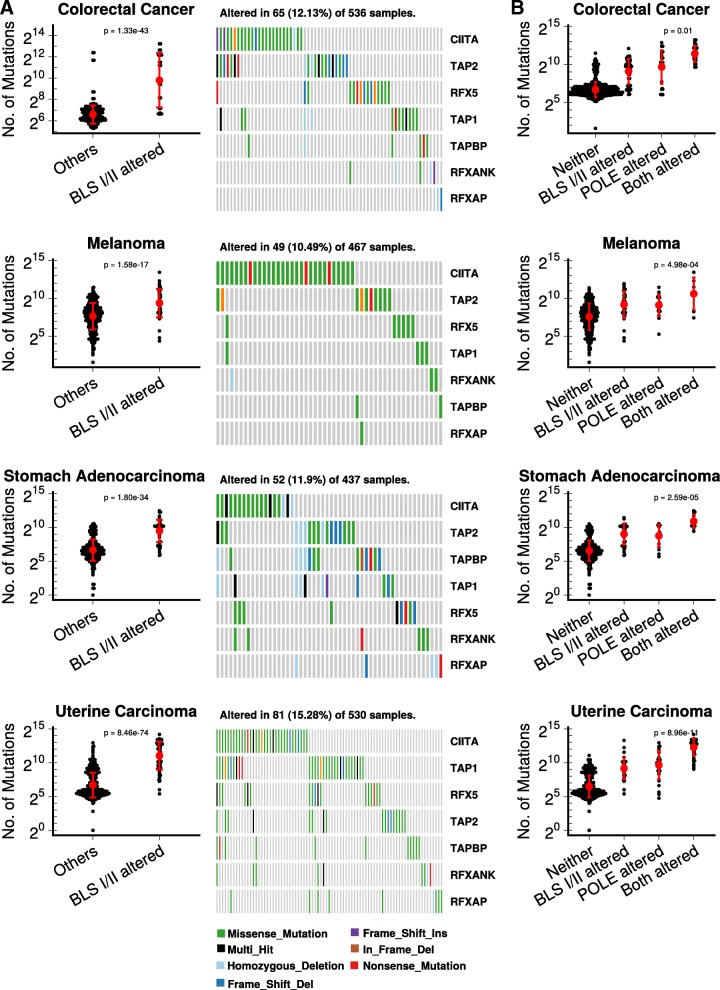
BLS-altered primary human tumors demonstrate high TMB in the context of POLE-mutator phenotypes. **a**) Left panels demonstrate association of BLS-altered tumors for each TCGA dataset (colorectal, melanoma, gastric/stomach, and uterine). BLS-alterations considered were nonsynonymous variants and deep deletions. The types of mutations are shown in the oncoprint on the right panels for each tumor type: light blue (homologous deletion), green (missense mutation), black (multi hit), blue (frame shift deletion), purple (frame shift insertion), brown (in frame deletion), and red (nonsense mutation). **b**) Association of TMB with the presence of a BLS class I/II alteration, a POLE mutation, or the combination of both. All comparisons were highly significant by negative binomial model test

Next, four TCGA tumor databases with a sizeable population of BLS-altered tumors (colorectal, melanoma, gastric and uterine) were selected. Intriguingly, within each database, tumors harboring BLS mutations were strongly associated with a higher TMB (*p* = 1.42e-17 to 8.82e-75 for all comparisons; Fig. 1a) compared to non-BLS-altered, suggesting that putative disruption of MHC-I or MHC-II antigen presentation may eliminate or reduce the requirement for immune-editing of the tumor by permiting the generation of neoantigens without an immune-mediated consequence.

Recently, proofreading domain mutations in polymerase-epsilon (Polε/*POLE*) have been described as associated with high TMB, immunogenic phenotypes, and response to immunotherapies [11, 12, 21]. Consistent with our hypothesis, BLS-altered tumors acquire greatly-increased TMB in the context of a *POLE*-deficient ‘mutator phenotype’ [21] (Fig. 1b). In all 4 tumor types tested, both *POLE-*mutant and BLS-mutant tumors had elevated TMBs. However, tumors with mutations in both *POLE* and BLS genes had exceptionally high mutation rates (*p* ≤ 0.01 for all tumor types compared to *POLE-*mutant alone), consistent with the notion that within a mutator-phenotype background, disruption of BLS genes may permit the acquisition of higher TMB without necessity of immunoediting.

**Fig. 2:**
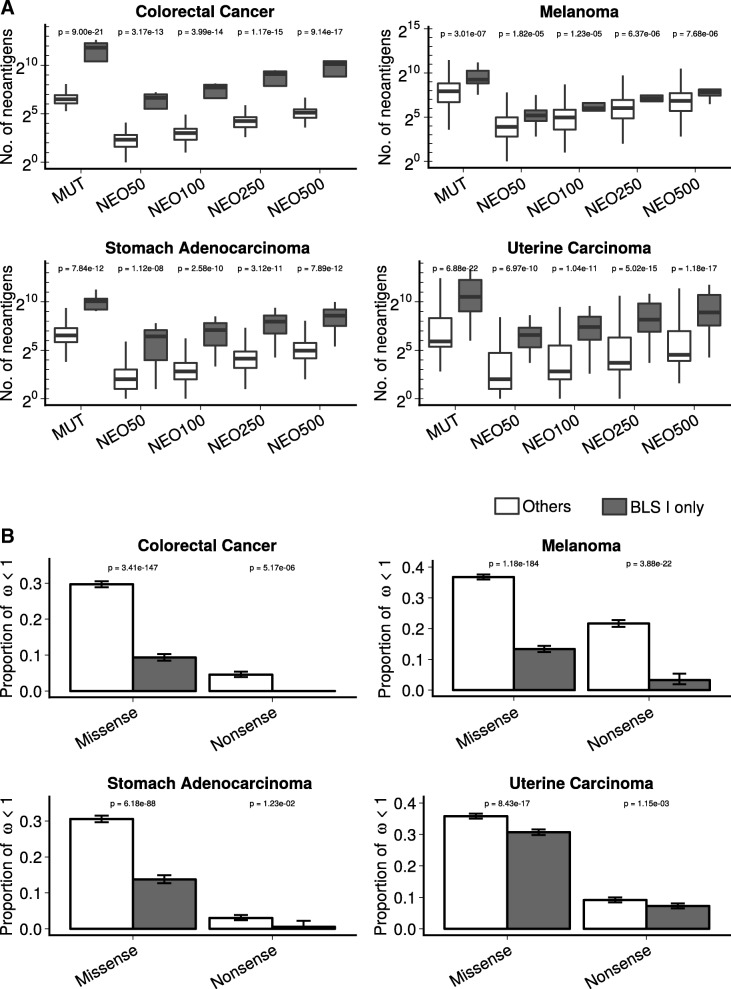
BLS altered tumors demonstrate disproportionally high neoantigen rates consistent with lack of immunoediting. **a**) TMB and neoantigen load across predicted binding affinity cutoffs in 4 cancer types. Type I BLS altered tumors demonstrated higher TMB and higher neoantigen rates across all cutoffs applied: 50 nM (NEO50), 100 (NEO100), 250 nM (NEO250), 500 nM (NEO500). **b**) Proportion of neoantigens carrying genes with *ω* (dN/dS ratio) < 1 between Type I BLS altered tumors and the Normal samples. dN/dS based on missense mutations and nonsense mutations were separately calculated and shown. Error bars show the 95% confidence interval

Not all mutations bind efficiently to patient-specific MHCs, and thus computational methods based on HLA-haplotype and known binding affinities are required to improve prediction accuracy for putative neoantigens. By in silico prediction of MHC class I specific neoantigens (which is more precise than MHC-II epitope prediction) using transcriptomic and whole-exome next generation sequencing data from TCGA, we asked whether BLS type I altered tumors within this cohort demonstrated a greater degree of predicted neoantigens than non-BLS type I altered counterparts (Additional file 2: Data Set). For both TMB and at each cut off of predicted binding affinity for neoantigens (50-500 nM) (Additional file 3: Table S1), we observed a strong association with BLS type I altered tumor status in all four cancer types (Fig. 2a).

Finally, we tested whether BLS type I altered tumors disproportionally develop putative neoantigens with reduced purifying selection, potentially due to disrupted immunoediting. Using a specifically designed method adapted from molecular evolution theory for cancer somatic evolution, we determined the selection pressure of neoantigen-carrying genes in different cohorts by calculating dN/dS ratios, a classic indicator for positive or purifying selection detection [22] (Additional file 3: Table S2). A consistent pattern of positive selection in majority of the neoantigen carrying genes were observed in all 4 cancer types as reported previously for the somatic mutations in all TCGA projects [22]. Interestingly, among the significantly, positively selected genes, a Type I BLS gene -*TAP2,* which encodes an ABC-transport protein required for transport of class-I peptides into the endoplasmic reticulum [23], was identified as a particular driver in colorectal cancer (*q* = 8.6e-4). In general, purifying selection showed stronger pressure on neoantigen carrying genes in non-BLS-I altered samples comparing with the BLS-I altered samples (*p* values of proportion test for percentages of neoantigen carrying genes with *ω* < 1 in all between groups are all under 0.05; Fig. 2b and Additional file 1: Figure S2). Thus, BLS-I altered samples appear to have reduced purifying selection pressure for developing neoantigens, possibly due to reduced immunoediting restrictions.

The effects of BLS alterations on clonal mutation selection and therapeutic response to immunotherapies are a potential area of importance to study. There have been several studies where data on somatic mutation load/neoantigen load have been collected on/post immunotherapy in patients [24–26]. We identified the cases with BLS I/II mutations in post therapy tumors, and compared the mutation load or neoantigen load, if available, with the cases without BLS mutations.

In the Riaz et al. cohort [25], which examined tumor mutational evolution during anti-PD-1 therapy, only 3 patients carried BLS gene mutations in their on-therapy tumors, two of which carried BLS type-II mutations in *CIITA*, and responded to anti-PD-1 therapy. Since the neoantigens predicted are MHC-I related, only one patient (who experienced stable disease [SD]) with a BLS type-I mutation in *TAP2* gene was appropriate to evaluate the change, which was not substantially different compared with all SD patients without BLS mutations.

For the Roh et al. cohort [21], exome sequencing data from longitudinal tissue samples from metastatic melanoma patients treated with sequential immune checkpoint blockade (CTLA-4 blockade followed by PD-1 blockade at time of progression) were available. As shown and described in Additional file 1: Figure S3A, this dataset contained two BLS type-I altered patients and five BLS type-II altered patients. Based on the limited information of the BLS altered cases in this dataset, there is a trend that BLS-altered patients retained high mutation load during or post immunotherapy, particularly in non-responders. However, these are highly exploratory data and conclusions cannot be drawn.

Finally, in the Le et al. cohort [15], mutation profiles and clinical information of 30 patients with mCRC treated with anti-PD-1 therapy, including a substantial proportion that were mismatch repair-deficient/microsatellite-instable, were investigated. There were 7 cases of BLS-altered tumors, which demonstrated higher mutation load, consistent with our prior data (Additional file 1: Figure S3B). However, all patients with BLS mutations were also microsatellite-instable in this dataset, which have an exceptionally high rate of response to immunotherapy [13, 26] (Additional file 1: Figure S3C). As such, 6 out of 7 BLS-altered cases were responders, complicating purifying selection analysis, as tumor response is typically accompanied by a substantial contraction of the tumor mutation burden due to elimination of tumor cells. No differences in outcomes were observed in this small population (data not shown). Since MSI is a major factor influencing genome stability, dysfunction of antigen presentation may be muted by the diversity and frequency of mutations generated in MSI tumors. This could lead to field-effects on anti-tumor immunity and immunoediting, or a substantial response to MHC-I restricted antigens (in BLS type-II-altered tumors, or vice versa). In brief, MSI status seems to predominate over mutation load in this case and may overcome the influences of BLS gene alterations, but it is difficult to conclude this without a larger sample size.

## Conclusions

In conclusion, we provide in silico support for the hypothesis that rare functionally-disruptive alterations in BLS genes may permit acquisition of high tumor mutation burdens, particularly in the context of mutation-prone genetic backgrounds such as *POLE* mutants. Furthermore, after adjusting for mutation burden, there was a significant gain in predicted neoantigens arising from BLS-altered tumors versus other tumors due to weakened constraints of purifying selection. These data support further investigation into the functionality of BLS alterations in cancer both molecularly and in clinical outcomes. Importantly, however, such investigations must include a detailed functional analysis, particularly in cases where the effect of the somatic alteration is unclear, such as SNVs.

Whether or not defects in BLS-associated genes are sufficient to drive immune evasion and their influence on immunotherapy outcomes remains to be more thoroughly explored. It is currently unclear in such cases whether activation of inflammation pathways (e.g. through Type I or II interferon activation/stimulation, such as STING activation or others) could overcome somatic defects in antigen presentation. The answers to such questions will likely need to be explored in murine models and may be subject to the type of alteration and clonality within the tumor cell population. Furthermore, clonal evolution studies of metastatic tumors harboring BLS-like alterations could offer additional insights into their role in immunoediting and driving immune evasion in certain contexts. Future work should extend these findings toward models testing causative association (e.g. mutator-phenotype murine cell lines harboring defects in BLS genes and testing acquisition of tumor mutations over time), as well as their impact on immunotherapy outcomes in larger analyses, which has already been demonstrated with *B2M* loss in some longitudinal studies [17, 26].

## Methods

### Genomic databases

Genomic variants and gene expression data, clinical factors (e.g. MSI status in colorectal tumors) and total mutational burden were accessed via the GDC Data Portal (https://portal.gdc.cancer.gov/). Copy number variation information was accessed from the Broad Institute TCGA GDAC (http://firebrowse.org/, GISTIC2 version, [27–30]). Data from immunotherapy-treated patients were accessed through their associated supplementary data files [24, 25].

### Neoantigen prediction

Patients specific HLA class I genotype was predicted using the whole exome sequencing data from matched normal tissue with OptiType [31]. GDC VCF files from the Mutect2 pipeline and the reads count based expression profiles for each case were achieved. Only non-synonymous SNPs or Indels were kept for somatic mutation load calculation and for neoantigen prediction. Raw reads count was transformed to transcript per million (TPM) as described in Bo et al. [32]. Combined with patient specific HLA genotypes, non-synonymous mutation list, and gene expression profile, MHC class I specific binding neoantigens were predicted in MuPeXI [33]. Binding affinity of MHC class I with candidate peptides was evaluated with netMHCpan 4.0 [34]. Mutated peptides with IC50 score < 500 nm while the according normal peptides with IC50 score > 500 nm were considered as candidate neoantigen peptides [35].

### Screen for selection signal

We used *dNdScv*, a set of maximum-likelihood dN/dS methods designed to quantify selection in cancer and somatic evolution [22], to study the selection pressure of the neoantigens in our study. dN/dS is the normalized ratio of non-synonymous to synonymous substitutions and widely used for selection detection in molecular evolution theory [36]. dNdScv utilized a modified dN/dS measurement, as described in Martincorena et al. [22], for the analysis of somatic mutations in cancer genomes with a few critical refinements, including a more comprehensive substitution model by considering the context-dependent mutational processes, addition of other non-synonymous mutation types beyond missense, careful elimination of germline variant contamination, and consideration of the variable mutation rate along the genome. This approach has been successfully applied for genome-wide scan of the somatic mutation selection patterns of different cancer types [22]. In Fig. 2b, samples were separated into “BLS I only” group if any non-synonymous mutations or deep deletions were occurred on *TAP1*, *TAP2* or *TAPBP* gene and “Others” if no non-synomymous mutations were detected in any BLS genes. For colorectal, gastric and uterine cancer, default parameters were used in *dNdScv*, while for melanoma, ultra-hypermutator mode was switched on due to large proportion of hypermutator tumors.

### Statistics

All statistical analyses were performed in R (www.r-project.org). For mutation load and neoantigen load comparison, negative binomial model was used to test the differences between different sample groups. A two-sample proportion test was used to test the difference of *ω* (dN/dS ratio) < 1 between BLS I only group and Others group and genes with *ω* = 0 were excluded. For all comparisons, *p* value was shown in plots. Bar graphs show mean ± standard error of the mean (SEM), unless otherwise stated in the figure legend.

## Additional files

Additional file 1: **Figure S1**. Rates and types of BLS alterations across human tumor datasets. A) cBioportal was utilized to access rates of deep deletion (GISTIC) and nonsynonymous genomic alterations in BLS genes across all datasets. B) Lollipop plot of individual mutations across human tumor datasets arranged by gene. **Figure S2**. ω (dN/dS ratio) of neoantigens carrying genes between Type I BLS altered tumors and the Normal samples. dN/dS based on missense mutations and nonsense mutations were separately calculated and shown. **Figure S3**. Changes in mutation burden and neoantigen load with immunotherapy. A) Sequencing data from longitudinal tissue samples from metastatic melanoma patients treated with sequential immune checkpoint blockade (CTLA-4 blockade followed by PD-1 blockade at time of progression) were extracted [24]. As shown in, there are two BLS I altered patients (Pt16: post-CTLA4_pre-PD1 and on-PD1; Pt22: on-CTLA4) and five BLS type-II altered patients (Pt21: post-CTLA4_pre-PD1; Pt26: post-CTLA4_pre-PD1 and post-PD1; Pt47: pre-CTLA4; Pt49: post-PD1; Pt53: pre-CTLA4 and on-PD1). Pt16 and Pt26 demonstrated relatively high and stable mutation load during anti-PD1 therapy. Pt22 had no BLS mutations before therapy but gained a new BLS type-I mutation during anti-CTLA4 treatment. Since this is a responder, total mutation burden was reduced dramatically, as expected. Only a single sample was available for both Pt21 and Pt49, however, the mutation load was relatively high in both cases. Interestingly, for Pt47, subclones harboring a BLS type-II mutation were eliminated during immunotherapy, accompanying the dramatic reduction in mutation load. Mutation load was slightly increased during the series of treatments for Pt53. B) Comparison of nonsynonymous mutation load between BLS mutated patients and control group in Le et al. 2017 Cohort. Only 30 mCRC patients with tumors post anti-PD1 therapy were investigated. BLS-altered tumors show significant or near significant higher mutation load than normal tumors, based on Wilcoxon test (*p* values are shown in plot). and C) Strata by MSI/MSS, the higher mutation load is associated with MSI. There are no significant differences between BLS-altered tumors and normal ones in MSI. Since all BLS-altered tumors are all MSI, we are unable to investigate how BLS mutations influence the mutation load without the interference of MSI. (PDF 674 kb)

Additional file 2: **Data Set 1**. Neoantigens predicted based on patient-specific HLA genotypes in colorectal, melanoma, gastric, and uterine cancers. (ZIP 63724 kb)

Additional file 3: **Table S1**. Mutation profiles of BLS genes in colorectal, melanoma, gastric, and uterine cancers. **Table S2**. ω (dN/dS ratio) and neutrality tests for selection signal of neoantigen carrying genes in BLS I only and Others group. Syn: synonymous mutation; mis: missense mutation; non: nonsense mutation. w: ω (dN/dS ratio); p: *P*-values for substitutions are obtained by Likelihood-Ratio Tests as described in (Martincorena et al., 2017); q: q-values are obtained by Benjamini-Hodgberg’s multiple testing correction. (XLS 12882 kb)
