# Supplementary material for: Tumor genomic alterations in severe-combined immunodeficiency bare-lymphocyte syndrome genes are associated with high mutational burden and disproportional neo-antigen rates
Source: J Immunother Cancer. 2019 May 7;7:123. doi: 10.1186/s40425-019-0584-2 (PMC6503546; doi:10.1186/s40425-019-0584-2)

Supplementary Figure 1

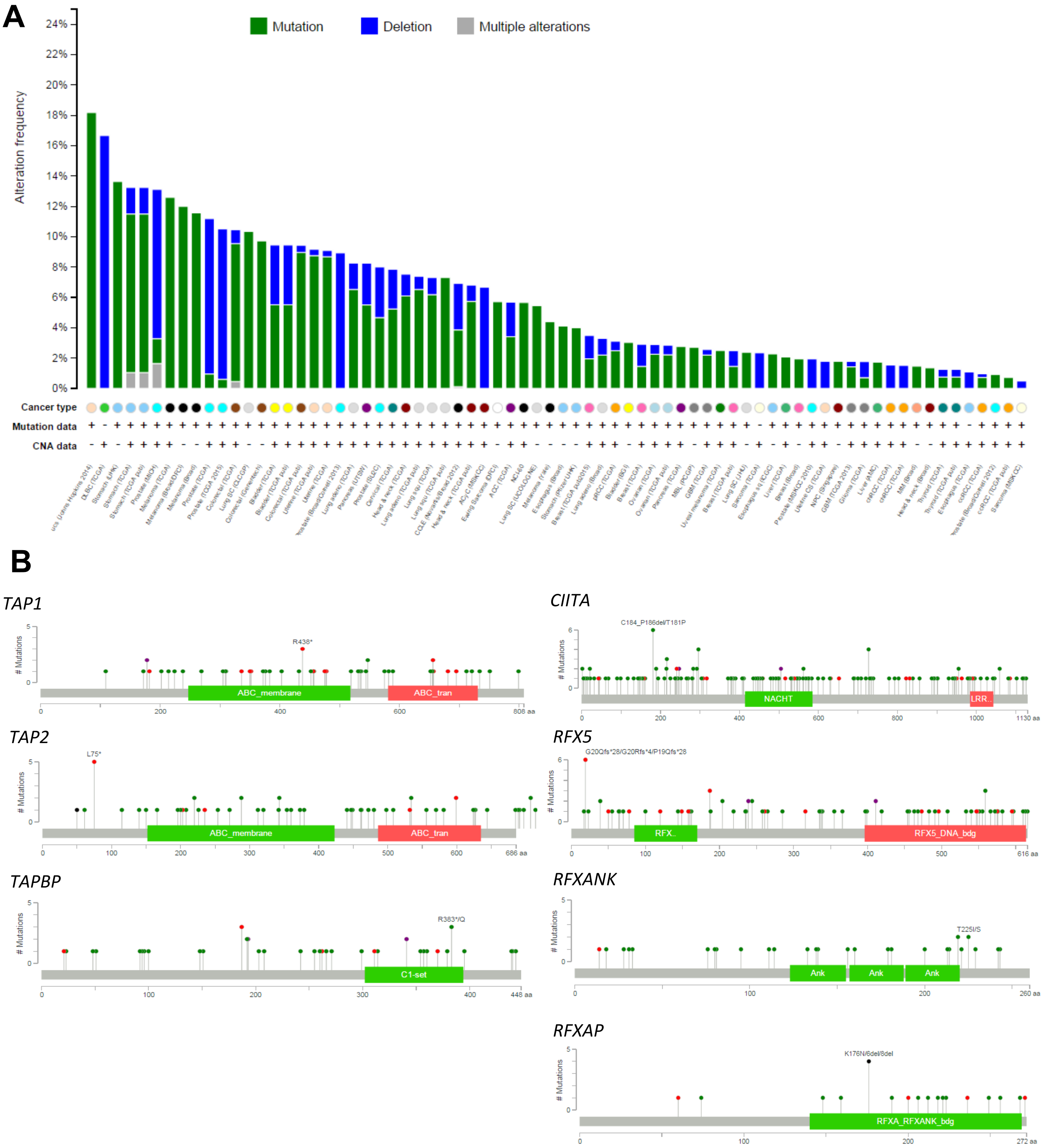

Supplementary Figure 2

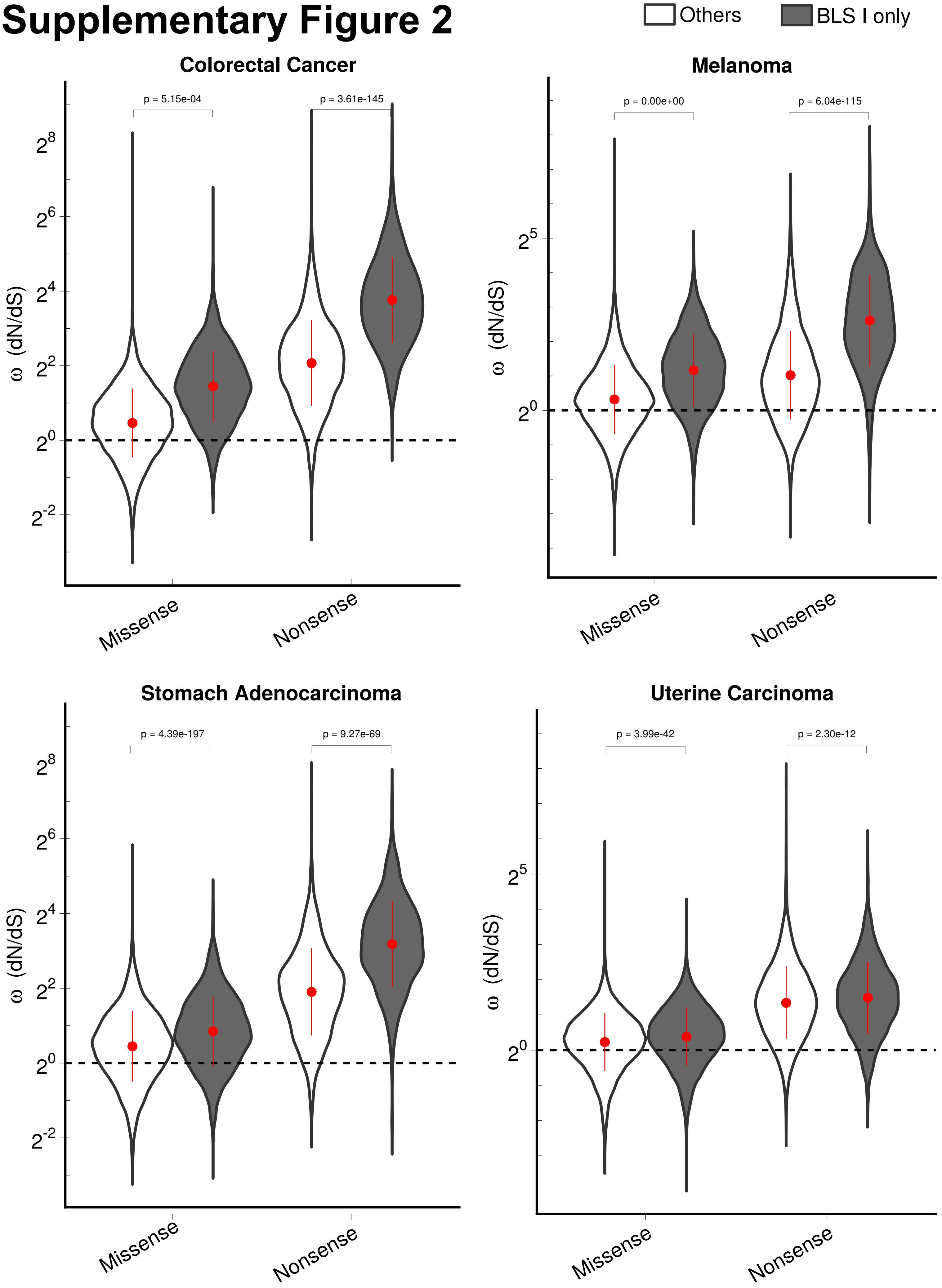

Supplementary Figure 3

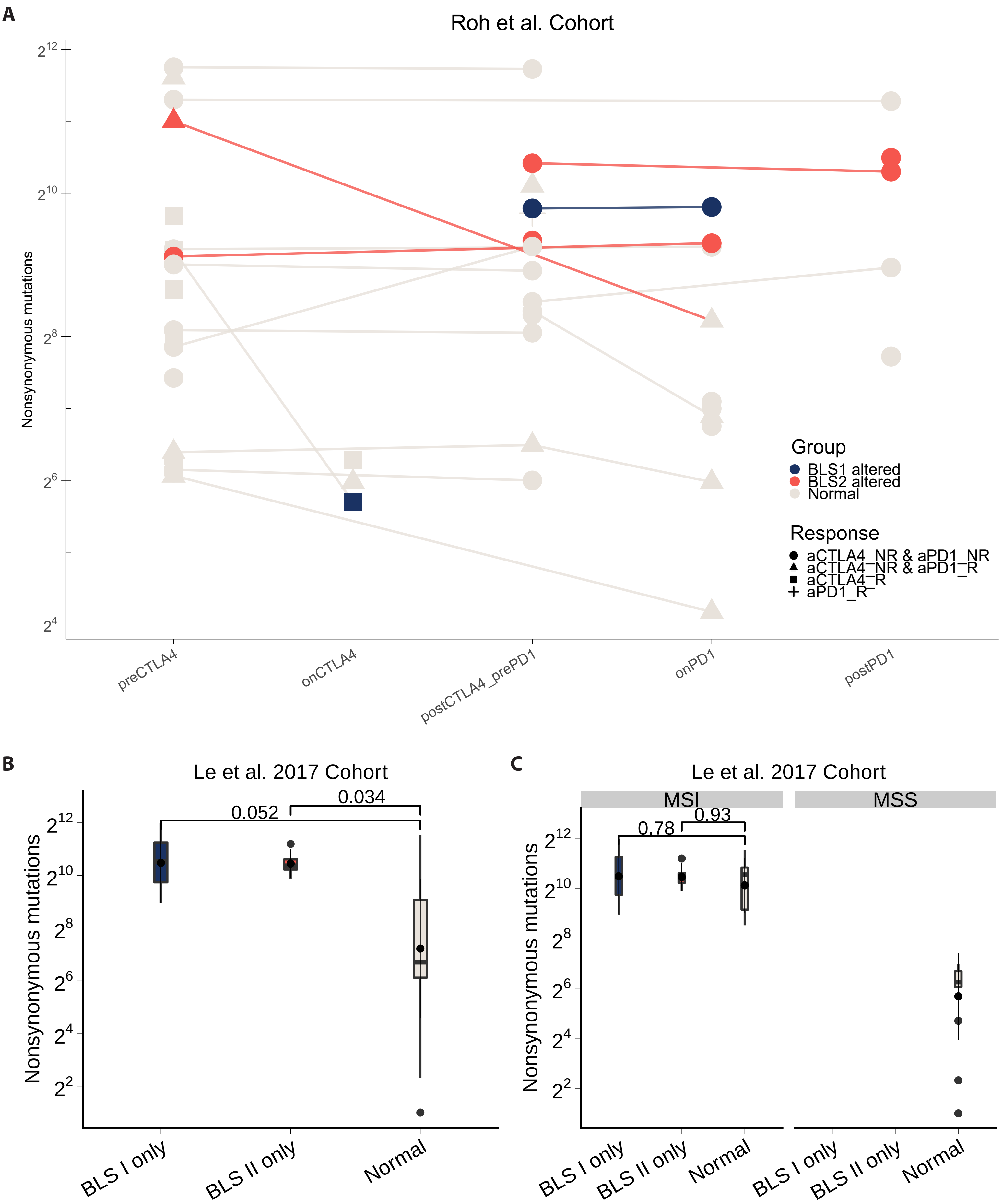

Supplement: Supplementary file 1 — Figure S1. Rates and types of BLS alterations across human tumor datasets. A) cBioportal was utilized to access rates of deep deletion (GISTIC) and nonsynonymous genomic alterations in BLS genes across all datasets. B) Lollipop plot of individual mutations across human tumor datasets arranged by gene. Figure S2. ω (dN/dS ratio) of neoantigens carrying genes between Type I BLS altered tumors and the Normal samples. dN/dS based on missense mutations and nonsense mutations were separately calculated and shown. Figure S3. Changes in mutation burden and neoantigen load with immunotherapy. A) Sequencing data from longitudinal tissue samples from metastatic melanoma patients treated with sequential immune checkpoint blockade (CTLA-4 blockade followed by PD-1 blockade at time of progression) were extracted [24]. As shown in, there are two BLS I altered patients (Pt16: post-CTLA4_pre-PD1 and on-PD1; Pt22: on-CTLA4) and five BLS type-II altered patients (Pt21: post-CTLA4_pre-PD1; Pt26: post-CTLA4_pre-PD1 and post-PD1; Pt47: pre-CTLA4; Pt49: post-PD1; Pt53: pre-CTLA4 and on-PD1). Pt16 and Pt26 demonstrated relatively high and stable mutation load during anti-PD1 therapy. Pt22 had no BLS mutations before therapy but gained a new BLS type-I mutation during anti-CTLA4 treatment. Since this is a responder, total mutation burden was reduced dramatically, as expected. Only a single sample was available for both Pt21 and Pt49, however, the mutation load was relatively high in both cases. Interestingly, for Pt47, subclones harboring a BLS type-II mutation were eliminated during immunotherapy, accompanying the dramatic reduction in mutation load. Mutation load was slightly increased during the series of treatments for Pt53. B) Comparison of nonsynonymous mutation load between BLS mutated patients and control group in Le et al. 2017 Cohort. Only 30 mCRC patients with tumors post anti-PD1 therapy were investigated. BLS-altered tumors show significant or near significant higher mutation l [file 40425_2019_584_MOESM1_ESM.pdf]
